# Non-Safety and Safety Device Sharp Injuries—Risk of Incidents, SEDs Availability, Attitudes and Perceptions of Nurses According to Cross-Sectional Survey in Poland

**DOI:** 10.3390/ijerph191811315

**Published:** 2022-09-08

**Authors:** Anna Garus-Pakowska, Mariusz Górajski, Piotr Sakowski

**Affiliations:** 1 Department of Nutrition and Epidemiology, Medical University of Łódź, 90-752 Łódź, Poland; 2 Faculty of Economics and Sociology, Department of Econometrics, University of Łódź, 90-214 Łódź, Poland; 3 IKM Pro Sakowska, Michałowska, Łyszkiewicz sp.j., 90-132 Łódź, Poland

**Keywords:** sharp injuries, needlestick injuries, nurses, safety-engineered devices, SEDs, non-safety devices

## Abstract

Sharp injuries are a serious issue among healthcare workers (HCWs). The aim of the study was to examine the frequency of sharps injuries among nurses (who have the most frequent contact with infectious material) when using devices with and without safety features, then to analyse the factors associated with such injuries and to compare the risk of injuries with safety engineered devices (SEDs) and non-safety engineered devices (non-SEDs). An online cross-sectional survey was completed between October 2021 and March 2022 by 280 nurses. The incidence of exposure to sharp injury during their professional life was 51.4%. The percentage of nurses experiencing a sharp injury in the year preceding the study was 29% and 9.6% for superficially and deep injury, respectively. Ampoules and conventional hollow-bore needles caused the most injuries (25.92% and 22.64% of nurses in the last year). Factors including sex (males), age and seniority (elderly), education (higher), work exhaustion and being left-handed were associated with the occurrence of conventional hollow-bore needle injuries. In the case of SEDs: age, seniority and right/left-handed were the most frequent risk factors associated with the occurrence of sharp injuries. SEDs injuries were much less frequent than non-SEDs. There was a significant difference between the risk of injuries with safety and non-safety needles, central cannulas and ampoules. Fisher’s exact test (*p*-value = 0.000) and positive Spearman’s rho statistics (0.2319, *p*-value = 0.0001) confirmed that in accredited hospitals, the availability of safety needles was higher. Almost half of the nurses (*n* = 115, 41.07%) stated that staff had little influence on the type of medical sharp instruments supplied. To reduce the risk of nurse injuries, access to medical devices with safe protection mechanisms should be ensured, the use of sharp instruments should be limited where possible, managers should consult nurses regarding the choice of safe devices, and training programs on the proper use of SEDs should be available.

## 1. Introduction

Sharp injuries (SIs) are a serious issue for the occupational health of healthcare workers (HCWs) all around the world. There are estimates providing numbers as high as above one million needlestick injuries annually in the European Union (EU) [1], and according to the Centers for Disease Control and Prevention, the estimated number of SIs among hospital-based healthcare staff in the USA is almost 400 thousand cases per year [2]. Apart from apparent health-threatening impact, they also constitute a high economic burden for national health systems. The successful claims made to NHS England (National Health Service) between 2012 and 2017 were valued at over 4 million GBP, and the estimates regarding the cost of SIs in Germany is 47 million EUR per year [3,4].

Occupational safety and health (OSH) issues have been precisely regulated by legislation, recommendations, and standards on the international and country levels. In the European realities, the EU directives must be transposed into the national legislation in the EU Member States. As a result, each EU country should shape its legislative regulations in a way that would impose a requirement of assuring healthy and safe working conditions in companies. More specifically, in each EU Member State, there should be specified duties and responsibilities of employers and workers, as well as general and specific actions contributing to achieving healthy and safe working conditions in each company.

One of the most important regulations of the European Union in the OSH area is the Framework Directive 89/391/EEC [5], which together with several individual directives shape the legislative principles for assuring health and safe working conditions. The Framework Directive sets up principles of health and safety at work, including obligations for employers (to ensure health and safety at the workplace, including risk management and control, provide information and training to workers) and employees (to be responsible for individual health at work as well as safety of co-workers and other persons present at the workplace, following procedures and instructions regarding occupational safety) and also bilateral obligations for consulting and agreeing on essential matters regarding OSH at the workplace [5,6]. Among the individual directives, there are Directives 2009/104/EC and 2000/54/EC regulating, respectively, minimum safety and health requirements for the use of work equipment by workers at work [7] and protection of workers exposed to biological agents at work [8]. In 2006, the European Parliament addressed the resolution to the European Commission regarding the protection of HCWs from blood-borne infections due to needlestick injuries (NSIs) [9], stressing the need for effective actions in that field. The urge for improvement in protection from SIs among HCWs has also been noticed and expressed in the Framework Agreement on prevention from SIs in the hospital and healthcare sector concluded by the European Hospital and Healthcare Employers’ Association and the European Federation of Public Services Unions [10]. The Agreement was later implemented by the Directive 2010/32/EU [11], which is a big milestone in assuring the occupational safety and health of healthcare staff.

Apart from the legislative tools, there are also international standards that relate to the issue of occupational safety and health, including Occupational Safety and Health Convention no. 155, which 74 countries have ratified (including 16 EU Member States), and Occupational Safety and Health Recommendation no. 164 by the International Labour Organization [12,13]. They refer to general principles of OSH, and although not particularly concerning needlestick injuries, they are shaping good practice in assuring a safe working environment. Many national authorities have recognized needlestick injuries as a serious occupational health issue and published information and guidelines on how to avoid them and what to do in case they happen [14,15,16,17].

HCWs in their daily work use many sharp tools. According to various sources, NSIs are the most common and nurses are the most at risk [18,19,20]. For the sake of HCWs health, new solutions are created that guarantee greater safety and protect against injury. These are safety engineering devices (SEDs). There is a large variety of SEDs available on the market. These include safe needles, safe cannula (intravenous, arterial and central), and safe scalpels. Basically, they are divided into two categories: active and passive. In active devices, the safety element demands activation by user, while in passive ones, it is activate automatically. Neither EU Directives nor national and international standards explicitly define which SEDs are the most effective, which has been the subject of scientific research and analysis over the years.

The general aim of the study was to analyse the frequency of SIs among nurses when using devices with and without safety features. The specific objectives included:

- To learn about the frequency of injuries among nurses,
- To identify the structure of injuries, taking into account type of injury, type of tool responsible for injury, as well as sociodemographic variables (i.e., seniority, place of work, education, gender, age),
- To evaluate access to safety tools in medical facilities,
- To assess respondents’ opinions on the use of safe medical tools in their profession,
- To compare the risk of injuries with safety and non-safety devices.

## 2. Materials and Methods

### 2.1. Study Design

The survey was conducted among nurses working in Poland in various healthcare institutions. We used a structured questionnaire containing 31 questions, covering thematic sections in line with the aim of the study:

(1) We assessed the frequency of injuries—questions about contact with potentially infectious material, injuries with selected safety and non-safety instruments during 12 months preceding the study;
(2) Questions about SEDs—SEDs availability, feedback on use, training, and views on whether nurses can influence SEDs purchasing decisions;
(3) Questions concerning sociodemographic variables of the nurses (e.g., age, gender, work experience, education or specialization) and hospital organization (type of hospital, number of beds, location, accreditation—means as external methods of ensuring high-quality services, etc.).

The survey was conducted online between October 2021 and March 2022. The questionnaire was anonymous and the respondents could stop completing the questionnaire at any time. The variable excluding participation in the study was a respondent performing a profession other than a nurse.

Sociodemographic variables concerning the respondent and those related to the workplace were included in the statistical analysis. Regarding access to safe tools and use of SEDs in daily work of nurses, we compared injury frequency among nurses who use SEDs with injury frequency among nurses who indicated difficult access to the SEDs or did not use SEDs.

### 2.2. Statistical Analysis

We used nonparametric tests for independence and correlation to study the existence of a stochastic relationship between variables describing the general population. We decided to use the following tests: the classical Fisher’s Exact test for count data and the Spearman rho and Kendall’s tau-b, which also indicated the trend of that relationship. We calculated the odds ratio of injures for several sociodemographic variables based on contingency tables and a logistic regression model. Besides, based on the number of injuries occurring in the 12 months preceding the study, we calculated the expected number of injuries for each device/per person/per year to compare the risk of injuries with SEDs and non-SEDs. We performed the paired Student’s *t*-test to check whether SEDs are safer in practice. As pairs, we have combined:

- Safety active needle + conventional needle,
- Safety passive needle + conventional needle,
- Safety active needle + safety passive needle,
- Safety intravenous cannula + conventional intravenous cannula,
- Safety arterial cannula + conventional arterial cannula,
- Safety central cannula + central cannula,
- Ampoule-syringe + ampoule.

The statistical computations were performed using STATA and Excel. The level of statistical significance was set at *p* = 0.05.

### 2.3. Ethical Concerns

The questionnaire collected no identifying personal data from the participants. The respondents were informed about the purpose of the survey, as well as about the possibility of stopping the survey at any time. According to Polish law and Good Clinical Practice regulations, the research, being an anonymous voluntary survey, did not require any approval from the Bioethics Committee.

## 3. Results

### 3.1. Characteristics of the Respondents in Terms of Sociodemographic Features

A total of 280 nurses participated in the study (261 female and 19 male). The structure of respondents in terms of sociodemographic features is presented in Table 1, while the variables characterizing the nurses’ workplace are presented in Table 2.

### 3.2. Frequency of Contact with Infectious Material and Frequency of Injuries

More than half of the surveyed nurses (*n* = 171, 61.07%) had contact with potentially infectious material even several times a day. Almost one in three reported such contact several times a week.

Throughout their professional career, 136 nurses (48.57%) have never been injured by a sharp medical instrument. Every second nurse (*n* = 144, 51.43%) declared that they had been injured with a sharp medical tool during their professional work.

In the 12 months preceding the study, 82 nurses (29.3%) were superficially injured, 43 of whom were injured at least several times. In the year preceding the study, 27 nurses (9.65%) suffered a deep injury, five of them (1.79%) several times.

### 3.3. Structure of Injuries over the Year Preceding the Study

In the year preceding the study, respondents most often injured themselves with a glass ampoule (25.92% of nurses among those who used such items) and a conventional hollow-bore needle (22.64%). The tools that were the least likely to cause injury in the last 12 months included safety central cannula (97.7% not injured), safety arterial cannula (96.95), and active and passive safety needles (94.44% and 92.86%, respectively) (Table 3).

In the case of needles, the conventional hollow-bore needle accounted for the greatest number of injuries. Overall, 22% of all nurses reported such NSIs during the 12 months preceding the study. Based on Fisher’s exact test, we confirmed that all analysed variables were associated with these injuries. Males showed about a five times higher chance of NSIs (OR = 4.92; 95% CI = 2.19–11.29). NSIs was positively associated with the age of nurses (OR = 4.92; 95%CI = 2.63–9.31) and with long work experience (OR = 5.46; 95% CI = 2.58–11.88). Work exhaustion was also positively associated with NSIs (OR = 1.78; 95% CI = 1.13–2.83). Nurses with higher education and those with specialization had a greater chance of being injured (OR = 2.74; 95% CI = 1.09–7.17 and OR = 1.86; 95% CI = 1.17–2.96, respectively). Right-handed nurses were injured significantly less frequently than left-handed nurses (OR = 0.31; 95% CI = 0.18–0.54).

In case of SEDs, injuries were much less frequent. Over 92% of nurses confirmed that they were not injured by a SEDs while working in the year preceding to the study.

There was a statistically significant difference between the needlestick injury (active and passive mechanism) and nurse age. Younger nurses were injured more frequently (OR = 0.98; 95% CI = 0.33–3.04 for active safety needles, and OR = 0.67; 95% CI = 0.25–1.77 for passive safety needles), but the relationship was not linear (*p*-value from Fisher’s exact test: 0.046 and 0.002). The incidence of needlestick injury was highest in nurses with fewer number of years in the profession (*p*-value from Fisher’s exact test: 0.065 for active safety needles, and *p* = 0.071 for passive safety needles). In case of the safety intravenous and arterial cannula, age (OR = 0.21; 95% CI = 0.06–0.65 for intravenous, and OR = 0.1; 95% CI = 0.02–0.43 for arterial safety cannula) and seniority (OR = 0.21; 95% CI = 0.07–0.67 for intravenous, and OR = 0.11; 95% CI = 0.02–0.44 for arterial safety cannula) influenced the chance of injury. Among the safe tools, men were more likely to get injured when using safety active needles (*p* = 0.099). For all SEDs, right-handed nurses were injured significantly less frequently than left-handed nurses (*p* = 0.078 for safety active needles, and *p* < 0.05 for other SEDs).

Generally, we found that age and seniority were the most frequent risk factors associated with the occurrence of sharp injuries (SEDs and non-SEDs) (Table 4).

Nurses primarily indicated the lack of skill in using such equipment (32%), patient movement (30%), and inattention (23%) as causes of injury with SED tools. Nurses also indicated technical problems with use of SED, which they believed contributed to the injury: safety mechanism closed, but incorrectly/not completely (7%) and safety guard closed, but then loosened (5%).

### 3.4. Access to Safety Tools in Medical Facilities

More than half of the respondents did not have access to the following types of safe medical equipment in their workplace:

- Safety hollow-bore needle (active and passive) (46.42%, *n* = 130);
- Safety intravenous cannula (58.92%, *n* = 165);
- Safety arterial cannula (69.28%, *n* = 194);
- Safety central cannula (59.64%, *n* = 167);
- Ampoule-syringes (56.07%, *n* = 157);
- Needle-free valves for safe access to the infusion set (63.57%, *n* = 178).

Fisher’s exact test (*p*-value = 0.345) and Spearman’s rho statistics (0.0366, *p*-value = 0.5417) confirmed that safety needles were available in hospitals and other medical facilities. There was no difference between the availability of safety needles. However, Fisher’s exact test (*p*-value = 0.000) and positive Spearman’s rho statistics (0.2319, *p*-value = 0.0001) confirmed that in accredited hospitals, the availability of safety needles was higher.

Almost half of the nurses (*n* = 115, 41.07%) stated that staff had little influence on the type of medical sharp instruments supplied, and the main purchase criterion was its price. In addition, 37 people (13.21%) replied that management did not control the quality of the safe medical equipment purchased or did not take into account the opinion of staff on this matter. Only every tenth nurse (*n* = 35, 12.5%) clearly confirmed that the hospital management considers the staff’s opinion on safe equipment used and takes this criterion into account when purchasing equipment.

### 3.5. Opinions on SEDs

When asked what safe medical equipment there was for them, the majority of nurses indicated the answer—“it’s a needle or a blade with a built-in mechanism protecting against risk of stabbing” 81.43% (*n* = 228). The answer “it is a medical tool that protects against injury in 100%” was chosen by 9.29% of the respondents (*n* = 26), and 3.21% of the nurses (*n* = 9) did not know what safe medical equipment was.

The differences between active and passive mechanisms of operation of safety equipment were known to 185 respondents (66.07%), while 33.93% of nurses (*n* = 95) did not know such differences.

According to the respondents, the most important features which should be characterized by safe medical equipment are: ease of use 68.21% (*n* = 191), durability 60.35% (*n* = 169), resistance to damage 51.78% (*n* = 145). Low price is a feature mentioned the least frequently, only by 15% (*n* = 42) of the respondents.

Training on use of safe medical equipment at work was carried out only among 40% of respondents (*n* = 112). Fisher’s exact test (*p*-value = 0.047) and positive Spearman’s rho statistics (0.1734, *p*-value = 0.0624) confirmed that training courses were more available in hospitals. Additionally, within hospitals Fisher’s exact test (*p*-value = 0.003) and positive Spearman’s rho statistics (0.1734, *p*-value = 0.0036) confirmed that training courses were more available in accredited hospitals than in hospitals without accreditation.

In the opinion of 37.86% (*n* = 106) of the nurses, the use of safety needles was very convenient and guaranteed safety during use. The conventional needle was chosen by 11.43% (*n* = 32) of the nurses instead of the safe one, and 4.64% (*n* = 13) did not like to use the SED. The respondents mentioned as the main reasons for not using safe medical equipment: poor access in the workplace (*n* = 126, 45%), and lack of training on use of safe needles (*n* = 56, 20%). The advantages of using SED were: reducing stress and anxiety (*n* = 86, 30.71%), simplifying everyday nursing duties (*n* = 52, 18.57%).

Additionally, we asked for an opinion on whether SED is really safer according to nurses. Interestingly, almost every third nurse (*n* = 76, 27.14%) replied that, according to them, the risk of injury was similar when using SED and non-SED devices. Every fifth nurse (*n* = 52, 18.57%) expressed an opinion that safe equipment is adjusted only to the manual needs/capabilities of right-handed people.

### 3.6. Comparison the Risk of Injuries with SED and Non-SED Devices

Besides, based on the number of injuries occurring in the 12 months preceding the study (Table 3), we calculated the expected number of injuries for each device/per person/per year to compare the risk of injuries with SEDs and non-SEDs. We found that the expected injury rate of SEDs was relatively low, below 0.1154 (Table 5).

Table 6 comprises the risk of injury (by device) measured by the average number of injuries in a year. Based on the paired Student’s *t*-test, there was a significant difference between SEDs and non-SEDs. The safety passive hollow-bore needle was safer than the conventional needle (diff = 0.48; *p*-value < 0.001), and the safety active hollow-bore needle was safer than the conventional needle (diff = 0.44; *p*-value < 0.001). Surprisingly there were no significant differences between the risk of injuries with an active and passive safety needles (diff = −0,02; *p*-value = 0.2172). Moreover, there was a significant difference between the risk of injuries with safety and non-safety central cannulas and ampoules. However, we found no difference in the risk of injury with safety and non-safety intravenous and arterial cannulas (Table 6).

## 4. Discussion

Due to the nature of their work, nurses have frequent contact with the patient, and thus with potentially infectious material (in our study 61%, several times a day). The incidence of exposure to sharp injury during their professional life was 51.4%. The percentage of nurses experiencing a sharp injury in the year preceding the study was 29% and 9.6% for superficial and deep injury, respectively. It is similar to some authors, e.g., Germany 28.7% and Saudi Arabia 22.2% [21,22], but less than others, e.g., Greece 74.1% and Ethiopia [20,23]. Among sharp wounds, many authors argue that needles and glass ampoules are the most dangerous tools [24,25]. Our research also found that these two devices cause the greatest number of injuries (25.92% nurses injured with glass ampoule in a year preceding the study and 22.64% with conventional hollow-bore needle).

There are many risk factors for SIs. Cui et al. showed that seniority and education influence the risk of injury [26]. These factors also turned out to be important in our study. In the Cui et al. survey, job category, title, department and training programs were also associated with the occurrence of SIs. Another risk factor for sharp injury may be working for more than 8 h per day [27]. Nurses who work long hours are often tired, and chronic fatigue can turn into emotional exhaustion, which can then lead to burnout. Both our and Cho et al.’s study confirmed that emotional exhaustion increases the risk of injury [28]. Additionally, we confirmed that left-handed people are more likely to be injured with sharp tools.

In the face of frequent HCWs injuries, tools with mechanisms protecting against injury have appeared on the market. By definition, a safe device has a design that prevents SIs before, during or after use thanks to built-in safety elements. In Italy, the injury rate/100,000 needles used has decreased after the introduction of the SEDs regulations [29]. A reduction in the injury rate from the use of SEDs has also been found in Japan [30]. The successful implementation of safe tools has been confirmed in US studies, where the high effectiveness of SEDs has been demonstrated. The number of injuries decreased by two-thirds for safety phlebotomy devices and more than 80% for safety I.V. catheters [31]. We also confirmed that SED injuries were much less frequent than non-SEDs. However, surprisingly, we did not show a difference in the risk of injury with active and passive safe needles, the greater effectiveness of which in protecting against injury is confirmed by various authors [32]. It seems to us that further studies, on a larger number of participants, should be conducted, and preferably a prospective multicentre study, showing the use of the number of tools and the number of wounds at the same time.

Nurses reported difficulties in accessing SEDs and a low level of training. Nurses would also like to influence the decisions managers make about purchasing SEDs. Some authors indicate, for example, problems with the activation of the safety mechanism [33]. We also confirmed it, which may suggest that there is still too little access to passive needles on the market [34,35]. Our risk analysis confirmed that age, seniority and being left-handed are a risk factors associated with the occurrence of SIs with SEDs, whereas nurses primarily indicated the lack of skill in using such equipment (32%), patient movement (30%), and inattention (23%) as cause of injury with SED tool.

Although safe tools do not exclude the risk of injury (but significantly reduce it), their use can also be considered from an economic point of view. Reports show annual numbers of NSIs at levels of over 100 thousand in Italy and almost 400 thousand in the USA, with around 50% underreporting rate [2,36]. Data from the United Kingdom, Germany, Spain and France also demonstrate a high burden of sharps injuries [37,38,39]. Studies of economic analysis show that the cost of single needlestick injury varies due to different circumstances and reaches values from several hundred to almost 1700 International US dollars (calculated for the year 2015) [36]. Other estimates regarding the annual cost of needlestick injuries provide numbers from 5 to 7 million EUR (Germany, Italy, France, Spain), through 300 million GBP in the United Kingdom, up to almost 600 million USD in the USA [39]. These are undisputable high costs for healthcare systems that can be to a certain level diminished by undertaking certain preventive measures. Use of SEDs is an example of such measures, allowing for significant savings. Estimated value of savings arising from using safe needles in the USA reaches around 40–50% [39,40]. Less optimistic cost-wise is a study from Belgium, showing estimated savings for a 420-bed hospital in a five-year period, resulting from use of SEDs, at the level of 12%; however, this includes the cost of acquisition of SEDs and not only savings from avoided NSIs [41]. Calculating costs of SIs and economic benefits arising from using SEDs is a complicated process with numerous variables, making simple cross-country comparison or comparison of results between different studies not easy, if possible at all. Nevertheless, research provides sound evidence for beneficial economic effects of using SEDs, allowing for reduction of the economic burden for healthcare systems arising from needlestick injuries.

Our results revealed that SIs are a serious issue among the Polish nurses, who are often subject to such incidents. Our study only included nurses, but all workers who come into contact with used needles and other medical sharps may be exposed to infectious material. Therefore, protective measures should cover nurses, but also doctors, laboratory workers, dentists, cleaners, etc. It is worthy noticing that assuring access to SEDs at the workplace is a substantial factor increasing workplace safety by reducing the risk of infection acquired through exposure to biological factors due to sharp injury. In each healthcare facility, risk management should be cautiously executed with proper implementation of the hierarchy of controls approach, particularly taking into account the appropriate choice of safe medical equipment and information and training on their use. Experience and training are important factors in limiting health threats resulting from NSIs. From the workplace safety perspective, dialogue and consultations between management and staff on the choice of SEDs, taking into account HCWs opinions regarding specific pieces of equipment, should have priority over selection of the SEDs-to-be-purchased based merely on the price aspect. In the concept of injury prevention, we can distinguish several levels—equally important. The first is injury prevention, such as the need to facilitate access to the SED described here, training in the use of safe tools, etc. Administrative control measures such as preparing a written statement of the health and safety policy, ensuring adequate staffing, proper organization of hospital work, easy access to sharps containers, and implementing an incident reporting system are also important [42]. In the prevention of infections, the most important role is played by prophylactic vaccinations (hepatitis B) and the implementation of post-exposure prophylaxis [43].

Our study had some limitations. As in all questionnaire surveys, we were unable to verify the truthfulness of the answers. It is also not a representative sample for the entire community of nurses in Poland, however, as a cross-sectional study, it gives appropriate conclusions to improve the safety situation of HCWs, and indicates the problems faced by nurses when using sharp tools. Furthermore, as we mentioned in the methodology section, based on the declared number of injuries occurring in the 12 months preceding the study we calculated the expected number of injuries for each device/per person/per year. We calculated the risk of injury for a group of nurses who used a given tool with different frequency. This allowed us to estimate the average frequency of use of the tool. However, in the future, we propose to calculate the expected number of injuries, taking into account the frequency of using a given tool. Such a question should be included in the survey questionnaire, and even better, a prospective study in which we could observe the number of procedures performed with the use of specific tools and at the same time observe the number of possible SIs. Unfortunately, such a study is long and much more expensive than a questionnaire study.

## 5. Conclusions

To reduce the risk of HCWs infection, access to medical devices with safe protection mechanisms should be ensured. The risk of injury with SEDs cannot be ruled out, but our study found that this is significantly lower than with conventional instruments. In every hospital premises, an assessment of the risk of injury should be performed and the use of needles and other sharp instruments should be limited where possible (e.g., using needleless drug delivery devices). In conjunction with the above recommendations, managers should consult nurses regarding the choice of safe devices and should not be guided by the low price of the product when purchasing. Training on the proper use of tools and how to deal with an injury is also very important. Modern security systems for medical tools provide for employee training in accordance with the principle that if well-trained personnel uses safe methods of work, security measures become more effective.

## Figures and Tables

**Table 1:** Characteristics of the included nurses (N = 280).

Variable	Frequency (*n*)	Percentage (%)
Age (years)		
<29	90	32.14
30–39	70	25.00
40–49	74	26.43
>50	46	16.43
Seniority (years)		
<5	68	24.29
5–10	53	18.93
10–20	78	2786
>20	81	28.93
Education		
Diploma	31	11.07
Bachelor of nursing/midwifery	131	46.79
Master	110	39.29
Doctor	8	2.86
Specialization		
Yes	112	40.00
Emotional exhaustion		
Yes	126	45.00
Right-handed/Left-handed		
Right-handed	236	84.29

**Table 2:** Characteristics of the workplace of the respondents.

Variable	Frequency (*n*)	Percentage (%)
Workplace		
hospital	257	91.79
primary healthcare	11	3.93
outpatient specialized care	9	3.21
other (laboratory, dialysis centre, primary school)	3	1.07
Type of hospital		
province	75	27.30
district (county)	54	19.93
clinical	51	18.82
municipal	54	19.93
institute	16	5.90
private	7	7.75
Department		
surgical	155	58.27
non-surgical	96	36.09
emergency	15	5.64
Number of hospital beds		
<100	50	17.86
101–300	82	29.29
301–500	83	29.64
501–1000	30	10.71
>1000	7	2.50
No/do not know	28	10.00
Accreditation		
yes	171	61.07

**Table 3:** The incidence of injuries with selected tools during the year preceding the study.

	Frequency of Injury						The Percentage of Those Who Never Injured Themselves among Those Who Used the Tool
Tool	Never *n* (%)	Not Applicable Because Did Not Use the Tool *n* (%)	Once *n* (%)	2–3 Times *n* (%)	4–5 Times *n* (%)	>5 *n* (%)	
Suture needle	204 (74.45)	40 (14.60)	14 (5.11)	10 (3.65)	2 (0.73)	4 (1.46)	87.18
Conventional hollow-bore needle	205 (73.74)	13 (4.68)	36 (12.95)	13 (4.68)	2 (0.72)	9 (3.24)	77.36
Safety (passive) hollow-bore needle	182 (65.47)	82 (29.50)	9 (3.24)	3 (1.08)	1 (0.36)	1 (0.36)	92.86
Safety (active) hollow-bore needle	170 (61.59)	96 (34.78)	6 (2.17)	3 (1.09)	-	1 (0.36)	94.44
Infusion needle	207 (74.19)	53 (19.00)	11 (3.94)	2 (0.72)	1 (0.36)	5 (1.79)	91.59
Pen needle	202 (72.40)	60 (21.51)	11 (3.94)	5 (1.79)	-	1 (0.36)	92.24
Dialysis needle	202 (72.92)	58 (20.94)	6 (2.17)	4 (1.44)	3 (1.08)	4 (1.44)	92.24
Ampoule-syringe	233 (83.51)	17 (6.09)	16 (5.73)	4 (1.44)	6 (2.15)	3 (1.08)	88.93
Scalpel	154 (55.40)	110 (39.57)	8 (2.88)	1 (0.36)	-	5 (1.80)	91.67
Ampoule	203 (72.76)	5 (1.79)	39 (13.98)	21 (7.53)	6 (2.15)	5 (1.79)	74.08
Intravenous cannula	250 (89.61)	18 (6.45)	4 (1.43)	3 (1.08)	1 (0.36)	3 (1.08)	95.78
Safety intravenous cannula	168 (60.22)	103 (36.92)	6 (2.15)	-	1 (0.36)	1 (0.36)	95.45
Arterial cannula	208 (74.55)	62 (22.22)	5 (1.79)	1 (0.36)	1 (0.36)	2 (0.72)	95.85
Safety arterial cannula	159 (57.19)	114 (41.01)	2 (0.72)	1 (0.36)	1 (0.36)	1 (0.36)	96.95
Transfusion equipment	149 (53.41)	121 (43.37)	5 (1.79)	2 (0.72)	1 (0.36)	1 (0.36)	94.30
Central cannula	209 (74.91)	59 (21.15)	5 (1.79)	3 (1.08)	2 (0.72)	1 (0.36)	95.00
Safety central cannula	170 (60.71)	106 (37.86)	2 (0.72)	-	1 (0.36)	1 (0.36)	97.70

**Table 4:** Risk analysis of factors associated with the occurrence of sharp injuries with selected tools during the year preceding the study.

Variable	Fisher’s Exact Test, *p*-Value	Kendall’s tau-b	95% CI		Odds Ratio	95% CI		Odds Ratio, Logit Model	*p*-Value	95% CI	
	Safety (active) hollow-bore needle										
sex, ref. = female	0.099	0.125	−0.061	0.311	2.84	0.69	10.90	2.84	0.095	0.83	9.70
age, ref. ≤ 29	0.046	−0.043	−0.174	0.088	0.98	0.33	3.04	0.88	0.539	0.58	1.33
seniority, ref. < 5	0.065	0.015	−0.111	0.140	0.96	0.32	2.95	1.07	0.729	0.73	1.56
education, ref. diploma	0.164	0.124	−0.001	0.249		0.43		1.44	0.068	0.97	2.12
specialization, ref. = no	0.816	0.030	−0.113	0.172	1.21	0.45	3.26	1.21	0.684	0.49	3.00
work exhaustion, ref. = no	0.490	0.060	−0.079	0.199	1.48	0.54	4.14	1.48	0.410	0.58	3.75
right-handed, ref = left-handed	0.100	−0.131	−0.304	0.042	0.39	0.12	1.27	0.39	0.078	0.14	1.11
	Safety (passive) hollow-bore needle										
sex, ref. = female	0.689	0.015	−0.129	0.159	1.18	0.00	6.10	1.18	0.833	0.25	5.59
age, ref. ≤ 29	0.002	0.066	−0.086	0.218	0.67	0.25	1.77	1.25	0.262	0.85	1.83
seniority, ref. < 5	0.071	0.035	−0.106	0.175	0.65	0.25	1.73	1.08	0.679	0.76	1.53
education, ref. diploma	0.086	0.100	−0.025	0.224		0.52		1.32	0.112	0.94	1.87
specialization, ref. = no	0.520	0.046	−0.091	0.184	1.33	0.53	3.38	1.33	0.508	0.57	3.13
work exhaustion, ref. = no	1.000	0.004	−0.133	0.140	1.02	0.40	2.59	1.02	0.960	0.44	2.39
right-handed, ref = left-handed	0.017	−0.178	−0.350	−0.007	0.30	0.11	0.87	0.30	0.014	0.12	0.79
	Conventional hollow-bore needle										
sex, ref. = female	0.000	0.239	0.138	0.340	4.92	2.19	11.29	4.92	0.000	2.30	10.50
age, ref. ≤ 29	0.000	0.158	0.065	0.251	4.92	2.63	9.31	1.39	0.002	1.13	1.71
seniority, ref. < 5	0.000	0.124	0.031	0.217	5.46	2.58	11.88	1.36	0.004	1.10	1.67
education, ref. diploma	0.001	0.188	0.092	0.285	2.74	1.09	7.17	1.36	0.000	1.15	1.61
specialization, ref. = no	0.006	0.151	0.046	0.256	1.86	1.17	2.96	1.86	0.005	1.20	2.89
work exhaustion, ref. = no	0.011	0.141	0.037	0.246	1.78	1.13	2.83	1.78	0.009	1.15	2.76
right-handed, ref = left-handed	0.000	−0.240	−0.345	−0.135	0.31	0.18	0.54	0.31	0.000	0.18	0.53
	Suture needle										
sex, ref. = female	0.002	0.198	0.066	0.329	3.84	1.54	9.61	3.84	0.002	1.66	8.88
age, ref. ≤ 29	0.000	0.167	0.063	0.272	3.18	1.51	6.83	1.47	0.002	1.15	1.88
seniority, ref. < 5	0.056	0.120	0.019	0.222	2.54	1.20	5.48	1.31	0.024	1.04	1.66
education, ref. diploma	0.000	0.194	0.077	0.311	0.91	0.39	2.15	1.34	0.003	1.10	1.63
specialization, ref. = no	0.007	0.162	0.045	0.279	2.07	1.18	3.64	2.07	0.007	1.22	3.51
work exhaustion, ref. = no	0.001	0.192	0.080	0.303	2.43	1.36	4.38	2.43	0.002	1.40	4.21
right-handed, ref = left-handed	0.857	−0.012	−0.130	0.106	0.93	0.44	2.00	0.93	0.841	0.46	1.88
	Infusion needle										
sex, ref. = female	1.000	0.003	−0.122	0.128	1.03	0.22	4.10	1.03	0.960	0.28	3.77
age, ref. ≤ 29	0.000	0.163	0.061	0.266	9.81	2.22		1.57	0.005	1.14	2.15
seniority, ref. < 5	0.026	0.101	0.001	0.202	4.82	1.36		1.33	0.063	0.98	1.81
education, ref. diploma	0.000	0.343	0.246	0.440		1.25		2.52	0.000	1.74	3.66
specialization, ref. = no	0.000	0.274	0.161	0.386	4.83	2.15	11.12	4.83	0.000	2.26	10.33
work exhaustion, ref. = no	0.000	0.233	0.122	0.344	3.89	1.73	8.94	3.89	0.000	1.82	8.31
right-handed, ref = left-handed	0.014	−0.164	−0.308	−0.019	0.38	0.17	0.86	0.38	0.012	0.18	0.80
	Pen needle										
sex, ref. = female	0.000	0.273	0.098	0.447	5.94	2.15	16.33	5.94	0.000	2.37	14.87
age, ref. ≤ 29	0.128	0.119	0.007	0.231	2.85	0.90	10.08	1.45	0.047	1.00	2.08
seniority, ref. <5	0.038	0.107	0.003	0.211	2.40	0.75	8.51	1.42	0.057	0.99	2.04
education, ref. diploma	0.008	0.158	0.032	0.284	1.56	0.42		1.42	0.022	1.05	1.91
specialization, ref. = no	0.083	0.116	−0.012	0.245	1.99	0.87	4.57	1.99	0.080	0.92	4.27
work exhaustion, ref. = no	0.053	0.134	0.011	0.257	2.27	0.96	5.48	2.27	0.045	1.02	5.07
right-handed, ref = left-handed	0.037	−0.157	−0.310	−0.003	0.37	0.15	0.93	0.37	0.020	0.16	0.85
	Dialysis needle										
sex, ref. = female	0.000	0.293	0.146	0.440	6.98	2.62	18.89	6.98	0.000	2.84	17.18
age, ref. ≤ 29	0.000	0.273	0.171	0.375	4.22	1.63	11.54	2.06	0.000	1.51	2.81
seniority, ref. < 5	0.000	0.221	0.127	0.315	5.34	1.75	18.22	1.86	0.000	1.36	2.54
education, ref. diploma	0.000	0.215	0.100	0.329	2.78	0.76		1.50	0.001	1.19	1.90
specialization, ref. = no	0.071	0.113	−0.009	0.236	1.73	0.92	3.26	1.73	0.069	0.96	3.13
work exhaustion, ref. = no	0.000	0.310	0.210	0.410	6.01	2.67	13.93	6.01	0.000	2.80	12.89
right-handed, ref = left-handed	0.000	−0.283	−0.421	−0.145	0.23	0.11	0.47	0.23	0.000	0.12	0.44
	Intravenous cannula										
sex, ref. = female	0.717	0.026	−0.101	0.152	1.33	0.29	5.23	1.33	0.664	0.37	4.81
age, ref. ≤ 29	0.076	0.113	0.009	0.218	2.35	0.89	6.58	1.42	0.035	1.02	1.97
seniority, ref. < 5	0.806	0.050	−0.053	0.154	1.40	0.55	3.69	1.16	0.344	0.85	1.59
education, ref. diploma	0.000	0.220	0.108	0.332		0.88		1.80	0.000	1.31	2.48
specialization, ref. = no	0.213	0.075	−0.042	0.192	1.57	0.74	3.34	1.57	0.208	0.78	3.16
work exhaustion, ref. = no	0.000	0.345	0.275	0.416	42.43	6.09		42.43	0.000	5.72	314.58
right-handed, ref = left-handed	0.000	−0.297	−0.443	−0.151	0.17	0.08	0.38	0.17	0.000	0.08	0.36
	Arterial cannula										
sex, ref. = female	0.077	0.136	−0.061	0.334	3.86	0.75	17.76	3.86	0.057	0.96	15.54
age, ref. ≤ 29	0.043	0.109	0.024	0.194		1.09		1.58	0.102	0.91	2.74
seniority, ref. < 5	0.002	0.108	0.040	0.177		0.85		1.78	0.058	0.98	3.24
education, ref. diploma	0.000	0.133	−0.010	0.275		0.28		1.66	0.045	1.01	2.71
specialization, ref. = no	0.385	0.069	−0.066	0.204	1.79	0.52	6.28	1.79	0.309	0.58	5.52
work exhaustion, ref. = no	0.000	0.260	0.188	0.332		3.03		1.00			
right-handed, ref = left-handed	0.002	−0.237	−0.413	−0.061	0.16	0.04	0.56	0.16	0.002	0.05	0.49
	Safety intravenous cannula										
sex, ref. = female	0.606	−0.084	−0.115	−0.053	0.00	0.00	4.35				
age, ref. ≤ 29	0.015	−0.161	−0.302	−0.020	0.21	0.06	0.65	0.55	0.025	0.33	0.93
seniority, ref. < 5	0.023	−0.148	−0.288	−0.007	0.21	0.07	0.67	0.62	0.031	0.40	0.96
education, ref. diploma	0.015	−0.082	−0.192	0.029		0.37		0.85	0.395	0.59	1.23
specialization, ref. = no	0.072	−0.136	−0.255	−0.016	0.32	0.07	1.23	0.32	0.078	0.09	1.14
work exhaustion, ref. = no	0.312	0.085	−0.054	0.224	1.83	0.59	5.88	1.83	0.253	0.65	5.19
right-handed, ref = left-handed	0.022	−0.187	−0.376	0.003	0.26	0.08	0.90	0.26	0.016	0.09	0.78
	Safety arterial cannula										
sex, ref. = female	0.604	−0.085	−0.116	−0.053	0.00	0.00	4.61	1.00			
age, ref. ≤ 29	0.000	−0.177	−0.333	−0.022	0.10	0.02	0.43	0.49	0.022	0.27	0.90
seniority, ref. < 5	0.002	−0.192	−0.336	−0.048	0.11	0.02	0.44	0.49	0.010	0.28	0.84
education, ref. diploma	0.041	−0.055	−0.176	0.066		0.30		0.90	0.628	0.60	1.36
specialization, ref. = no	0.254	−0.102	−0.232	0.028	0.42	0.09	1.70	0.42	0.191	0.11	1.55
work exhaustion, ref. = no	0.590	0.044	−0.104	0.192	1.38	0.41	4.75	1.38	0.563	0.46	4.17
right-handed, ref = left-handed	0.007	−0.240	−0.445	−0.035	0.18	0.05	0.67	0.18	0.004	0.06	0.58
	Central cannula										
sex, ref. = female	0.058	0.131	−0.036	0.299	3.02	0.85	10.16	3.02	0.052	0.99	9.17
age, ref. ≤ 29	0.024	0.071	−0.050	0.193	1.65	0.60	4.75	1.27	0.189	0.89	1.82
seniority, ref. < 5	0.952	0.033	−0.081	0.147	1.32	0.48	3.84	1.10	0.562	0.79	1.55
education, ref. diploma	0.002	0.184	0.061	0.307	3.07	0.41		1.62	0.006	1.15	2.27
specialization, ref. = no	0.119	0.104	−0.024	0.231	1.87	0.81	4.34	1.87	0.112	0.86	4.05
work exhaustion, ref. = no	0.000	0.337	0.259	0.414	34.14	4.83		34.14	0.001	4.56	255.26
right-handed, ref = left-handed	0.000	−0.257	−0.419	−0.094	0.20	0.08	0.51	0.20	0.000	0.09	0.47
	Safety central cannula										
sex, ref. = female	1.000	0.000	−0.145	0.146	1.01	0.00	8.59	1.01	0.995	0.12	8.36
age, ref. ≤ 29	0.000	0.043	−0.133	0.220	0.45	0.13	1.59	1.22	0.448	0.73	2.02
seniority, ref. < 5	0.044	0.010	−0.151	0.172	0.46	0.13	1.64	0.99	0.969	0.63	1.56
education, ref. diploma	0.009	−0.081	−0.192	0.030		0.31		0.84	0.403	0.56	1.26
specialization, ref. = no	0.778	0.023	−0.123	0.169	1.20	0.34	4.18	1.20	0.758	0.39	3.71
work exhaustion, ref. = no	0.000	0.255	0.183	0.327		2.38					
right-handed, ref = left-handed	0.377	0.105	0.069	0.140		0.37					
	Ampoule										
sex, ref. = female	0.845	0.012	−0.091	0.115	1.09	0.47	2.52	1.09	0.821	0.50	2.37
age, ref. ≤ 29	0.002	0.177	0.086	0.268	2.28	1.37	3.80	1.44	0.000	1.18	1.75
seniority, ref. < 5	0.000	0.080	−0.015	0.174	0.94	0.57	1.57	1.16	0.109	0.97	1.39
education, ref. diploma	0.000	0.050	−0.047	0.147	1.48	0.71	3.13	1.08	0.291	0.93	1.26
specialization, ref. = no	0.451	−0.044	−0.146	0.058	0.83	0.53	1.30	0.83	0.399	0.55	1.27
work exhaustion, ref. = no	0.006	0.146	0.044	0.247	1.81	1.16	2.80	1.81	0.006	1.19	2.74
right-handed, ref = left-handed	0.040	−0.110	−0.213	−0.008	0.56	0.32	1.00	0.56	0.036	0.33	0.96
	Ampoule syringe										
sex, ref. = female	1.000	0.000	−0.145	0.146	1.01	0.00	8.59	1.01	0.995	0.12	8.36
age, ref. ≤ 29	0.000	0.043	−0.133	0.220	0.45	0.13	1.59	1.22	0.448	0.73	2.02
seniority, ref. < 5	0.044	0.010	−0.151	0.172	0.46	0.13	1.64	0.99	0.969	0.63	1.56
education, ref. diploma	0.009	−0.081	−0.192	0.030		0.31		0.84	0.403	0.56	1.26
specialization, ref. = no	0.778	0.023	−0.123	0.169	1.20	0.34	4.18	1.20	0.758	0.39	3.71
work exhaustion, ref. = no	0.000	0.255	0.183	0.327		2.38					
right-handed, ref = left-handed	0.377	0.105	0.069	0.140	0.37						
	Scalpel										
sex, ref. = female	0.586	−0.084	−0.122	−0.045	0.00	0.00	4.44				
age, ref. ≤ 29	0.000	0.145	−0.005	0.295	0.73	0.32	1.66	1.42	0.031	1.03	1.95
seniority, ref. < 5	0.009	0.076	−0.064	0.216	0.73	0.32	1.66	1.13	0.392	0.85	1.52
education, ref. diploma	0.019	0.157	0.029	0.286	5.63	0.76		1.38	0.020	1.05	1.81
specialization, ref. = no	0.113	0.116	−0.026	0.257	1.77	0.84	3.74	1.77	0.108	0.88	3.53
work exhaustion, ref. = no	0.001	0.241	0.119	0.362	3.92	1.60	9.88	3.92	0.001	1.70	9.02
right-handed, ref = left-handed	0.075	−0.131	−0.291	0.029	0.44	0.17	1.19	0.44	0.073	0.18	1.08
	Transfusion equipment										
sex, ref. = female	1.000	−0.073	−0.106	−0.040	0.00	0.00	6.63				
age, ref. ≤ 29	0.004	−0.021	−0.163	0.121	0.57	0.21	1.57	0.93	0.736	0.61	1.41
seniority, ref. < 5	0.076	−0.029	−0.185	0.128	0.44	0.16	1.20	0.90	0.558	0.62	1.29
education, ref. diploma	0.059	−0.060	−0.174	0.055		0.56		0.92	0.637	0.67	1.28
specialization, ref. = no	0.105	−0.130	−0.263	0.004	0.41	0.13	1.28	0.41	0.098	0.14	1.18
work exhaustion, ref. = no	0.649	−0.046	−0.196	0.103	0.76	0.28	2.02	0.76	0.547	0.31	1.86
right-handed, ref = left-handed	0.100	−0.138	−0.319	0.044	0.39	0.12	1.28	0.39	0.080	0.14	1.12

95% CI—Confidence Interval.

**Table 5:** Expected (average) number of injuries in a year per person by device.

Device	Average Number of Injury in a Year
Ampoule	0.5420
Conventional hollow-bore needle	0.4962
Suture needle	0.3077
Ampoule-syringe	0.2710
Dialysis needle	0.2443
Scalpel	0.2411
Infusion needle	0.1770
Intravenous cannula	0.1303
Transfusion equipment	0.1297
Pen needle	0.1279
Central cannula	0.1250
Safety hollow-bore needle (passive)	0.1154
Safety hollow-bore needle (active)	0.1083
Safety intravenous cannula	0.0938
Safety arterial cannula	0.0823
Safety central cannula	0.0718
Arterial cannula	0.0558

**Table 6:** Comparison of the risk of injuries with SED and non-SED devices.

Paired Student’s *t*-Test	Difference in Risk, Ref. = Row	*p*-Value
Conventional hollow-bore needle	Safety hollow-bore needle (passive)	
	0.48	0.0001
Conventional hollow-bore needle	Safety hollow-bore needle (active)	
	0.44	0.0001
Safety hollow-bore needle (active)	Safety hollow-bore needle (passive)	
	−0.02	0.21
Intravenous cannula	Safety intravenous cannula	
	0.06	0.1642
Arterial cannula	Safety arterial cannula	
	0.003	0.4705
Ampoule	Ampoule-syringe	
	0.29	0.0001
Central cannula	Safety central cannula	
	0.12	0.0054

## Data Availability

Not applicable.
